# A Novel Anastomosis Technique for Laparoscopic Pancreaticoduodenectomy: Case Series of Our Center's Experience

**DOI:** 10.3389/fsurg.2021.583671

**Published:** 2021-03-12

**Authors:** Pi-Jiang Sun, Yan-Hua Yu, Jian-Wei Li, Xi-Jun Cui

**Affiliations:** ^1^Department of Hepatobiliary Surgery, Weihai Central Hospital Affiliated to Qingdao University, Weihai, China; ^2^Department of Dermatology, Weihai Central Hospital Affiliated to Qingdao University, Weihai, China; ^3^Institute of Hepatopancreatobiliary Surgery, First Affiliated Hospital of Army Medical University, Chongqing, China

**Keywords:** laparoscopic pancreaticoduodenectomy, pancreaticojejunostomy, Blumgart, U-shaped suture through the pancreas, anastomosis of the pancreatic duct and the jejunal mucosa, pancreatic fistula

## Abstract

**Background:** Laparoscopic pancreaticoduodenectomy has developed rapidly in recent years. Postoperative pancreatic fistula is still the most dangerous complication of laparoscopic pancreaticoduodenectomy. Baumgart pancreaticojejunostomy is considered one of the safest anastomosis procedures, with low rates of pancreatic fistula. We modified Blumgart pancreaticojejunostomy and applied the modified procedure during laparoscopic pancreaticoduodenectomy. The modified procedure entailed a longitudinal U-shaped suture through the pancreas for anastomosis of the pancreatic duct and the jejunal mucosa.

**Methods:** We prospectively collected and retrospectively analyzed the data of 120 patients who underwent laparoscopic pancreaticoduodenectomy from January 2016. The total operative time, time for complete pancreaticojejunostomy, postoperative pancreatic fistula rate, postoperative delayed gastric emptying, postoperative bleeding, postoperative length of hospital stays, and mortality within 90 days after surgery were analyzed. An analysis of laparoscopic pancreaticojejunostomy compared with open pancreaticojejunostomy is also reported.

**Results:** In the laparoscopic pancreaticojejunostomy group, the average total operative time, the average time for complete pancreaticojejunostomy, and the average intraoperative blood loss were 271 min, 35.3 min, and 184 ml, respectively. The total postoperative clinically relevant pancreatic fistula rate was 9.2% (Grade B and C fistulas). The incidence rates of postoperative delayed gastric emptying and postoperative biliary fistula were ~2.5 and 1.7%, respectively. The postoperative bleeding rate was 0.83%, and the average postoperative indwelling time of the abdominal drainage tube was 7.3 days. The postoperative length of hospital stay was 10.8 days, and the mortality rate within 90 days after surgery was 0.83%. The rates of clinically relevant postoperative clinically relevant pancreatic fistula are comparable between laparoscopic and open surgery, there were no other severe postoperative complications in either group. The mean postoperative length of hospital stay was significantly shorter in the laparoscopic pancreaticojejunostomy group.

**Conclusion:** The modified laparoscopic-adapted Blumgart anastomosis simplifies and facilitates the creation of the pancreaticojejunostomy in laparoscopic pancreaticoduodenectomy. The rates of clinically relevant postoperative pancreatic fistula are comparable with those obtained by open surgery, and length of stay are shoter.

## Introduction

Pancreaticoduodenectomy is a classic surgical procedure for the treatment of benign and malignant tumors around the head of the pancreas, the lower common bile duct, the duodenum, and the ampulla (1). However, due to the complex anatomical relationship around the pancreas, the softness of the pancreas, the strong corrosiveness of pancreatic fluid, and the high surgical skill requirement, pancreaticoduodenectomy has been considered a high-risk procedure (2). Laparoscopic techniques have evolved since laparoscopic pancreaticoduodenectomy was reported in 1994 (3). Laparoscopic pancreaticoduodenectomy has been developed rapidly in various pancreatic surgical centers (4, 5), but laparoscopic pancreaticojejunostomy is more challenging than open pancreaticojejunostomy. Abdominal infection, bleeding, and even the life-threatening risk associated with pancreatic fistula are still considered difficult problems. To reduce the incidence of postoperative pancreatic fistula, pancreatic surgeons have invented a variety of approaches for pancreaticojejunostomy. However, no optimal approach is available for pancreaticojejunostomy. Since Blumgart anastomosis for pancreaticoenterostomy was first reported, it has become a well-accepted procedure among pancreatic surgeons and is considered one of the safest anastomosis procedures with low rates of pancreatic fistula after open pancreaticoduodenectomy (6, 7). We modified the Blumgart anastomosis for pancreaticoenterostomy according to Chen's longitudinal U-suture technique (8) and applied the modified approach to laparoscopic pancreaticoduodenectomy.

This article aims to describe our laparoscopic-adapted pancreaticojejunostomy for laparoscopic pancreaticoduodenectomy and to compare the results with open surgery.

## Materials and Methods

We began using the modified Blumgart pancreaticojejunostomy for laparoscopic pancreaticoduodenectomy in 2016. To date, 120 cases of laparoscopic pancreaticoduodenectomy have been performed using this anastomotic technique. We prospectively collected and retrospectively analyzed the patients' general clinical characteristics (age, sex, body mass index, pancreatic duct diameter, pancreatic texture, and histopathological diagnosis), surgical results (total operative time, the time for complete pancreaticojejunostomy, and estimated intraoperative blood loss), and postoperative results (amylase level on days 1, 3, and 5 after surgery, the number of days with an indwelling drainage tube, the incidence of postoperative delayed gastric emptying, the postoperative clinically relevant pancreatic fistula rate, postoperative length of hospital stay, and mortality within 90 days after surgery) (Table 1). All patients were informed about the possible advantages and disadvantages of laparoscopic surgery, and informed consent was obtained. Institutional review board approval for this study was obtained from our hospital.

**Table 1:** Demographics, operative characteristics and postoperative complications in 120 patients.

**Background characteristics**	
Sex (male/female), *n*	74/46
Age (years)	57.82 (26–78)
Diseases, *n*	120
Ampullary carcinoma	30
Distal cholangiocarcinoma	27
Carcinoma of the pancreatic head	25
duodenal papillary carcinoma	14
Adenocarcinoma of the duodenum	6
Solid pseudopapillary tumor	3
Pancreatic head adenoma	2
Intraductal papillary mucinous neoplasms	3
Pancreatic duct stones	5
Neuroendocrine tumor	4
Lymphoma	1
**Pancreatic parenchymal texture**, ***n***	
Soft	79
Hard	41
**Pancreatic duct diameter**, ***n***	
≥3 mm	72
≤3 mm	48
**Operative characteristics**	
Operation time (min)	271.4
Pancreaticojejunostomy time (min)	35.3
Conversion (*n*, %)	2(1.67%)
Operative blood loss (mL)	184
Required transfusion, *n* (%)	7(5.83%)
Hospital stay (days)	10.8
Postoperative complications, *n* (%)	
Peritoneal effusion, *n* (%)	11(9.2%)
Gastrointestinal hemorrhage, *n* (%)	1(0.83%)
Delayed gastric emptying, *n* (%)	3(2.5%)
Biliary fistula, *n* (%)	2(1.7%)
Abdominal hemorrhage, *n* (%)	1(0.83%)
Chylous fistula, *n* (%)	1(0.83%)
POPF, *n* (%)	
Biochemical leak	24(20%)
Grade B	10(8.3%)
Grade C	1(0.83%)
Re-operation, *n* (%)	1(0.83%)
Mortality, *n* (%)	1(0.83%)

## Operative procedure

### Patient Position and Trocar Distribution

**Figure 1:**
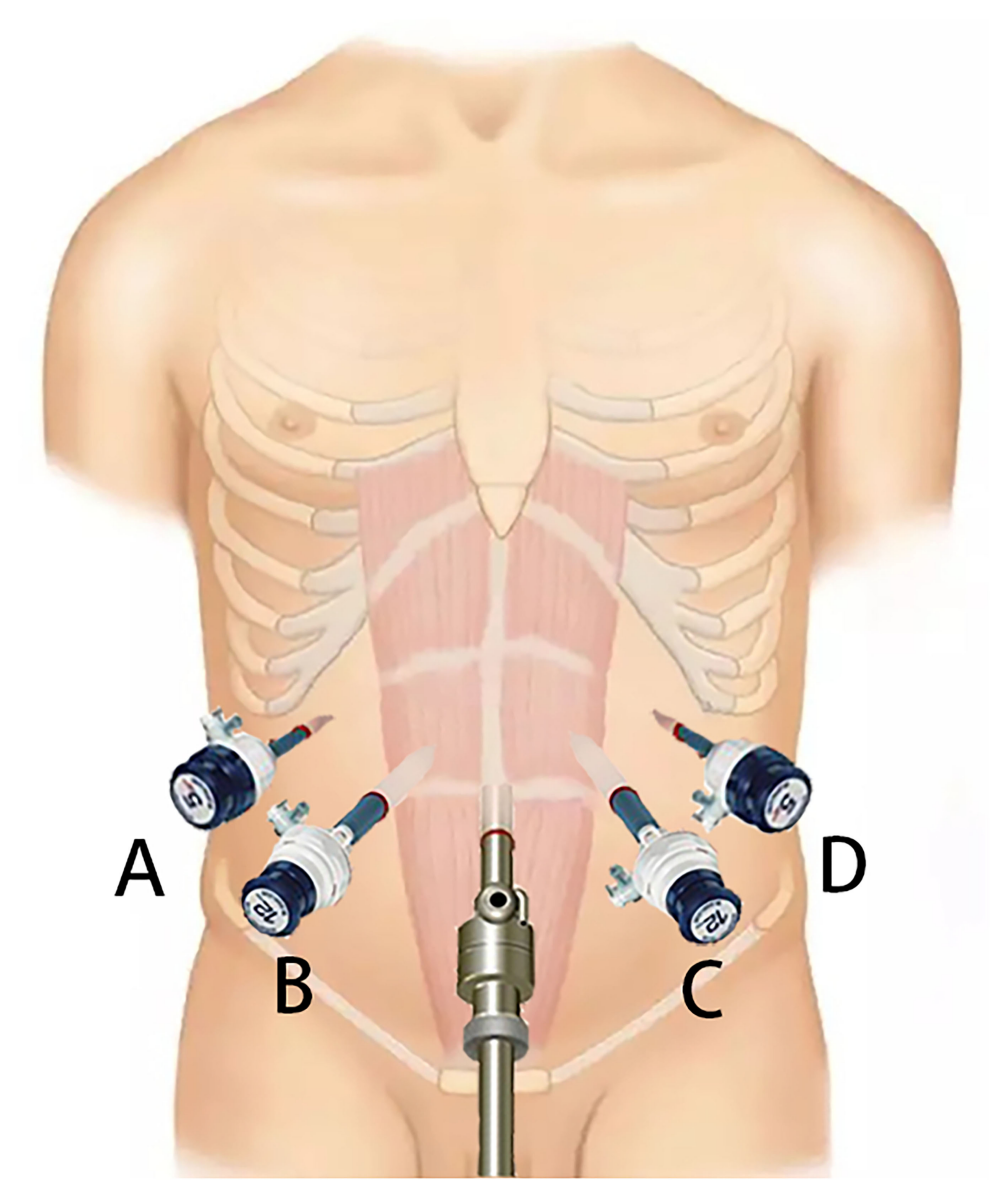
Placement of the trocars for Laparoscopic pancreaticoduodenectomy.

The patient is placed in the supine position, with the head higher than the feet (forming a 30-degree incline). Five trocars are inserted symmetrically in a “fan-shaped” pattern. After the specimen is removed, laparoscopic pancreaticojejunostomy is completed through the B and C trocars (Figure 1).

### Pancreaticojejunostomy Procedure

**Figure 2:**
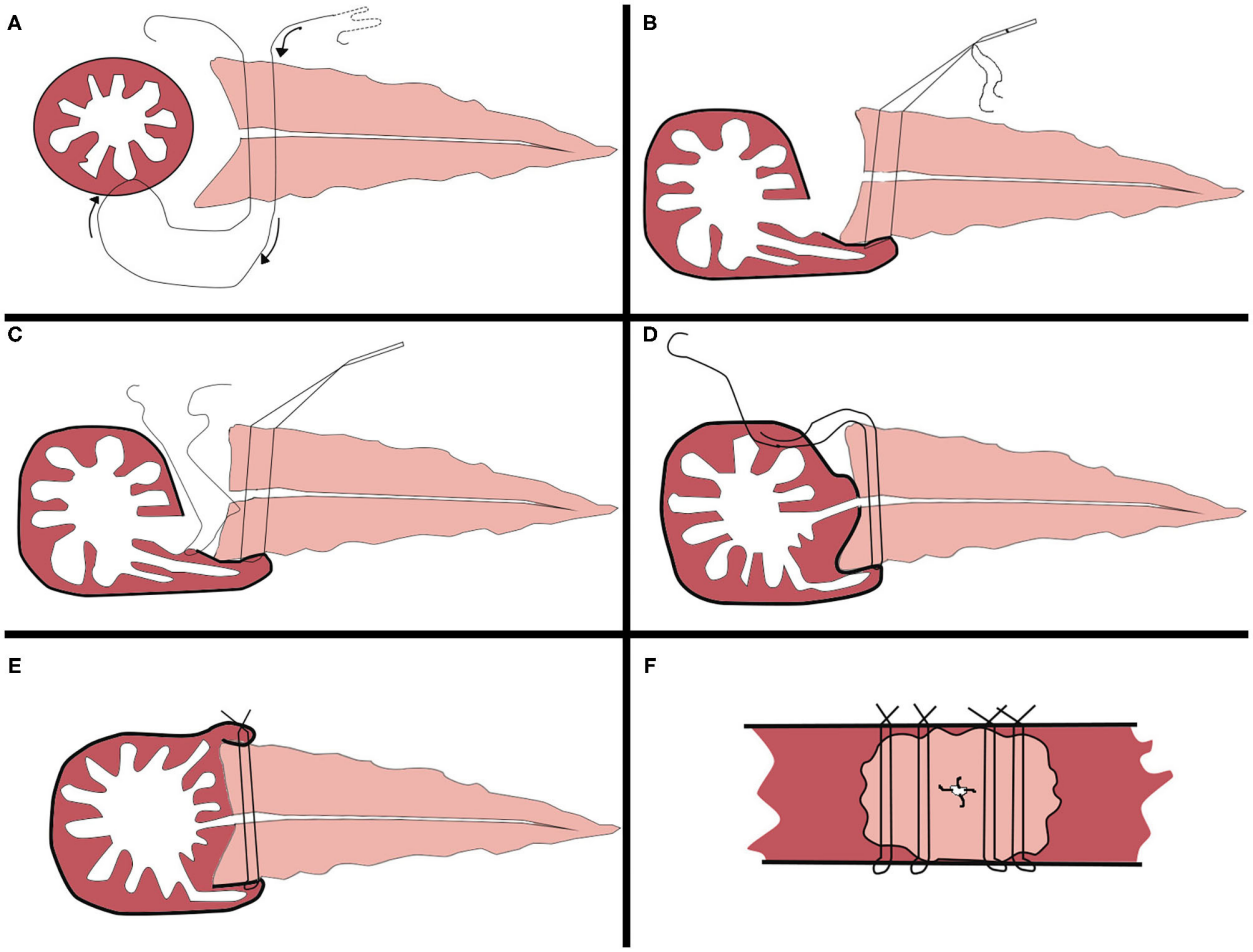
Mode chart of the modified Blumgart pancreaticojejunostomy procedure. **(A)** Placement of four interrupted penetrating U-sutures with 3-0 proline, large needle. **(B)** Approximation of the jejunal seromuscular layer. **(C)** Duct-to-mucosa anastomosis with four interrupted 5-0 PDS. **(D)** Suturing of the anterior wall of the jejunal seromuscular layer. **(E)** Tying of the knots on the jejunum one by one, longitudinal view of the completed anastomosis. **(F)** Transverse view of the completed anastomosis.

**Figure 3:**
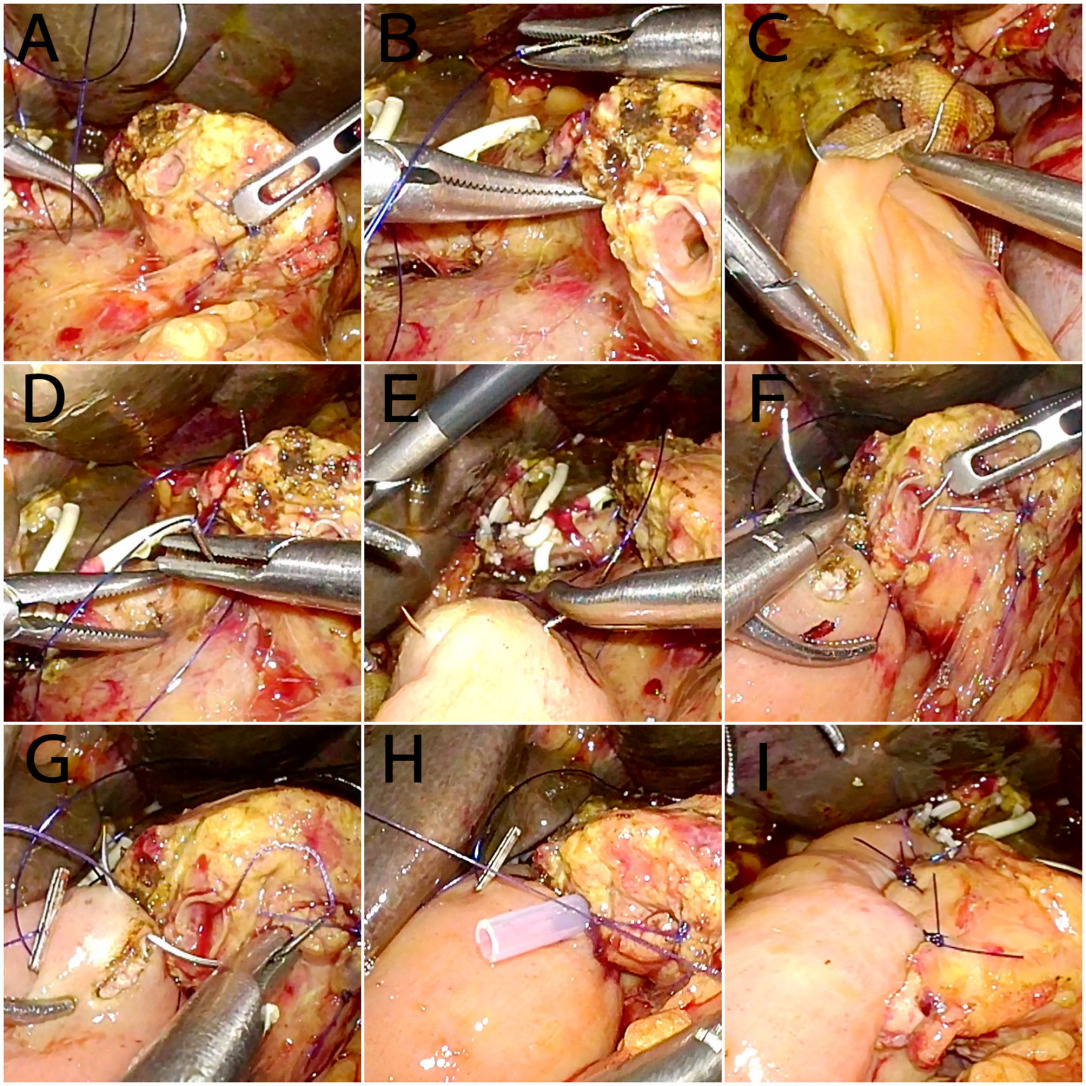
Intraoperative images of the modified Blumgart pancreaticojejunostomy procedure. **(A)** The specimen has been removed; pancreaticojejunostomy proceeds next. **(B)** At 1.5 cm from the edge of the pancreatic stump, a 3-0 large needle penetrates the pancreas. **(C)** Stitching of the posterior wall of the jejunum. **(D)** At 0.5 cm from the edge of the pancreatic stump (at the same level as the previous stitch), the needle penetrates the pancreas. **(E)** Stitching of the anterior wall of the jejunum. **(F,G)** Stitching of the pancreatic duct and jejunal mucosa. **(H)** An internal pancreatic stent is placed, and the duct-to-mucosa anastomosis is continued. **(I)** After suturing using 4–5 needles, all sutures were tied.

After the specimen is excised, at 1.5 cm from the incised end of the pancreas, the jejunal stump is introduced to the incised end of the pancreas through the anterior colon approach for anastomosis (**Figure 3A**). (1) A 3-0 large needle with a prolene suture vertically enters the pancreas from the ventral side of the pancreas at the site 1.5 cm from the edge of the pancreatic stump and extends out from the corresponding position on the dorsal side of the pancreas (**Figure 3B**). The needle then enters the posterior wall of the jejunum vertically to its long axis and is advanced 1 cm within the seromuscular layers (**Figure 3C**), and the needle then extends out and enters the pancreas at a site 0.5 cm from the edge of the pancreatic stump (at the same level as the previous stitch) through the pancreas from the dorsal side to the ventral side (**Figure 3D**). The suture appears as a U-shape (Figures 2A,B). (2) The anastomosis between the pancreatic duct and the jejunal mucosa is the same as traditional Blumgart anastomosis. An incision is made at a site 5 cm away from the edge of the jejunal stump and opposite to the mesenteric membrane (the corresponding position of the pancreatic duct). Interrupted 5-0 PDS sutures are used to perform an anastomosis between the whole layer of the pancreatic duct and the posterior wall of the jejunal mucosa (Figure 2C). The sutures are knotted with the suture end uncut. A supportive tube is placed in the pancreatic duct. Then, the uncut sutures are used to tie and secure the pancreatic duct to the supportive tube (Figure 3H). The extra sutures are cut. Then, interrupted 5-0 PDS sutures are continued to complete the suture between the whole layer of the pancreatic duct and the upper, lower, and anterior walls of the jejunal mucosa (Figures 3F,G). Then, the sutures are knotted. (3) The 3-0 prolene suture continues on the anterior wall of the jejunal seromuscular layer (corresponding to the suturing site of the posterior wall of the jejunum), and the sutures are not tied (Figures 2D, 3E). The same U-shaped suturing is performed at 4 or 5 sites with a 5-mm interval according to the width of the pancreas. Care should be taken to avoid damaging the main pancreatic duct. (4) The previously placed U-shaped prolene sutures are tied and knotted in succession (Figures 2E,F, 3I). Appropriate strength must be used for knotting to prevent cutting of the pancreas parenchyma due to an overtightened suture. After bilioenteric anastomosis completion and gastrojejunostomy, drainage tubes are placed above and below the pancreas-enteric anastomosis.

### Postoperative Management

On the 1st, 3rd, and 5th days after surgery, the amylase level in the drainage fluid of each drainage tube is examined. The gastric tube is removed on the 3rd day after surgery, and follow-up enhanced computed tomography (CT) of the upper abdomen is performed on the 5th day after surgery. The postoperative pancreatic fistula is diagnosed based on the diagnostic and grading criteria of the International Study Group of Pancreatic Surgery (ISGPS), i.e., after 3 days, the prospective amylase level in the drainage fluid is still 3-fold higher than the upper limit of the normal blood amylase level. According to the clinical imaging results of the patient, the pancreatic fistula is divided into three categories (grades A, B, and C). Grade A refers to a biochemical fistula, and clinically relevant pancreatic fistulas are defined as grade B or grade C pancreatic fistula. Delayed gastric emptying refers to gastrointestinal decompression through a nasogastric tube for more than 7 days. On the 5th day after surgery, if the amylase level of the drainage fluid is lower than three times the normal serum amylase level and follow-up enhanced CT of the upper abdomen shows no sign of abdominal fluid in the operative region, the drainage tube can be removed. If a pancreatic fistula or related complications occur, treatments can be initiated accordingly. In patients with poor drainage, an F18 double-lumen urinary catheter with the balloon removed should be used as a drainage tube to replace the original abdominal drainage tube on the 7th day after surgery. In patients with unclear drainage fluid, irrigation with saline can be performed using the indwelling urinary catheter. The patients are followed up to 3 month after discharge from the hospital.

### Study Design

All 118 patients treated by laparoscopic-adapted Blumgart anastomosis (LBA) (two patients who were converted to an open procedure were excluded) and 53 patients treated with open Blumgart anastomosis (OBA) were compared. For statistical analysis, the SPSS 25 software package was used. Nonparametric Mann–Whitney U and Fisher's exact tests were used to analyze quantitative and qualitative variables.

## Results

A total of 120 patients underwent laparoscopic pancreaticoduodenectomy with modified Blumgart pancreaticojejunostomy, including 74 males and 46 females. Of these patients, 30 patients were diagnosed with ampullary carcinoma, 27 with distal cholangiocarcinoma, 25 with carcinoma of the pancreatic head, 14 with duodenal papillary carcinoma, six with adenocarcinoma of the duodenum, five with pancreatic duct stones in the pancreatic head, three with a solid pseudopapillary tumor of the pancreas, two with pancreatic head adenoma, three with intraductal papillary mucinous neoplasms (IPMNs) of the pancreas, four with neuroendocrine tumor, and one with lymphoma. The average total operative time, the average time for complete pancreaticojejunostomy, and the average intraoperative blood loss were 271.4 min, 35.3 min, and 184 ml, respectively. Twenty-four (20%) patients were diagnosed with biochemical leak after surgery, and 10 (8.3%) patients were diagnosed with Grade B pancreatic fistula. One (0.83%) patient was diagnosed with Grade C pancreatic fistula. One case of postoperative biliary anastomotic fistula was reported, with an incidence rate of 0.83%, and three cases of postoperative delayed gastric emptying were reported, with an incidence rate of 2.5%. One case of postoperative abdominal hemorrhage occurred because the supportive tube in the pancreatic-intestinal anastomosis site penetrated the jejunum and caused bleeding. The patient was cured after reoperation for homeostasis. Postoperative peritoneal effusion was reported in 11 patients (9.2%), and the effusion subsided after negative pressure suction drainage. No patients required postoperative puncture for drainage. The average indwelling time of the drainage tube was 7.35 days, and the average postoperative length of hospital stay was 10.8 days. One patient died of bleeding due to a gastrointestinal anastomotic ulcer within 90 days after surgery, resulting in a 90-day mortality rate of 0.83%.

Table 2 shows the results of the comparison between laparoscopic pancreaticoduodenectomy and open pancreaticoduodenectomy. No differences between the groups were found in age, sex, parenchymal texture of the pancreas, and the diameter of the pancreatic duct. No differences were found in postoperative pancreatic fistula and severe postoperative complications between the two groups, but the postoperative hospital stay in the laparoscopic group was significantly shorter.

**Table 2:** Results of the study between laparoscopic pancreatic oduodenectomy (in laparoscopic group 2 patients converted were excluded) and open pancreaticoduodenectomy.

	**LPD**	**OPD**	***P***
*N*	118	53	
Age (years)^a^	58.4 (10.5)	55.1 (10.0)	0.06
**Sex^b^**			
Man	72	31	
Woman	46	22	0.75
**Pancreatic parenchymal texture**^**b**^			
Soft	77	31	
Hard	41	22	0.40
**Pancreatic duct diameter**^**b**^			
≥3 mm	47	25	
≤3 mm	71	28	0.37
Gastrointestinal hemorrhage^b^	1 (0.85%)	3 (5.66%)	0.054
Delayed gastric emptying^b^	3 (2.54%)	4 (7.55%)	0.13
Biliary fistula^b^	2 (1.69%)	2 (3.77%)	0.41
Abdominal hemorrhage^b^	1 (0.85%)	3 (5.66%)	0.054
Chylous fistula^b^	1 (0.85%)	0	1
POPF Grade B-C^b^	11 (9.32%)	7 (13.21%)	0.44
Length of stay(days)^a^	10.3 (5.5)	15.6 (4.5)	<0.01
Mortality <90 days^b^	1 (0.85%)	0	1

*LPD, laparoscopic pancreaticoduodenectomy; OPD, open pancreaticoduodenectomy*.

Data are presented according to:

a mean (SD);

b *n (%)*.

## Discussion

With advancements in surgical techniques and technology, pancreaticoduodenectomy has become safer, but the incidence of clinically relevant pancreatic fistula after pancreaticoduodenectomy is ~15%. Once a pancreatic fistula occurs, the mortality rate is ~16%. Currently, anastomosis of the pancreatic duct and jejunal mucosa and the “invaginated” anastomosis are the most widely used procedures in clinical practice (9). Peng et al. reported a bundled pancreaticojejunostomy procedure with a modified invaginated anastomosis in 150 consecutive patients, and no pancreatic fistula was reported; however, the mortality rate was ~3%. The main cause of death was pancreatic hemorrhage (10). However, subsequent foreign studies did not reproduce their results showing no pancreatic fistula formation (11–13). The incidence of pancreatic fistula was low using the U-suture technique for longitudinal invaginated pancreaticojejunostomy invented by the scholar Chen et al. (8). However, as an invaginated anastomosis, there is still a risk of bleeding directly caused by intestinal fluid corrosion at the stump of the pancreas. To date, anastomosis of the pancreatic duct and jejunal mucosa is still a preferred method by pancreatic surgeons (14). Blumgart pancreaticojejunostomy is a representative anastomosis of the pancreatic duct and jejunal mucosa, with a minimized risk of pancreatic fistula. However, the procedure has disadvantages, such as requiring more suturing and being a cumbersome laparoscopic operation. We used Chen Xiaoping's method of a longitudinal U-shaped suture through the pancreas as a reference to improve the outer layer suture of the Blumgart anastomosis and applied it to laparoscopic pancreaticoduodenectomy. We prospectively collected and retrospectively analyzed the clinical data and the prognoses of patients. Poves et al. reported a similar technique of laparoscopic-adapted Blumgart pancreaticojejunostomy, with a 15.4% incidence of postoperative pancreatic fistula. (15) In our study, the incidence of postoperative pancreatic fistula is 9.2%, which was lower than thant reported by Poves. Peng et al. also reported a laparoscopic-adapted pancreaticojejunostomy technique with a lower incidence of pancreatic fistula, about 3.8% (16). The clinically relevant pancreatic fistula rate in our study was close to but higher than that reported by Peng et al. This shows that our post-laparoscopic pancreatic fistula rate is acceptable compared to other studies. Comparison between open pancreaticojejunostomy and laparoscopic pancreaticojejunostomy has been also made. Posteriorly, a analysis was performed between 118 laparoscopic pancreaticojejunostomy and 53 open pancreaticojejunostomy. The rates of clinically relevant POPF are comparable between laparoscopic and open surgery, there were no other severe postoperative complications in either group, but the mean postoperative length of stay was significantly shorter in patients operated laparoscopically.

The advantages of this modified anastomosis are as follows. 1. The modified anastomosis requires less suturing, thus leading to reduced extravasation of pancreatic fluid from the pinholes and a more convenient laparoscopic operation. 2. The longitudinal U-shaped suture anastomosis through the pancreas can minimize shear forces of the suture during knotting, is associated with a lower risk of cutting the pancreas, and is also suitable for use in softer pancreases. 3. The modified suture is perpendicular to the long axis of the intestine. Compared with traditional Blumgart anastomosis, the modified suture is more conducive to the fit of the intestine and the stump of the pancreas, eliminating the dead space of the intestinal wall and the stump of the pancreas and thereby reducing fistula formation between the pancreatic duct and capillary pancreatic duct in the cross-section of the pancreas. The modified procedure can reduce the risk of bleeding at the stump of the pancreas. 4. The intestinal wall is overlapped on the ventral and dorsal sides of the pancreatic stump, covering the pinholes of the ventral and dorsal sides of the pancreas to reduce pancreatic fluid accumulation in the fistula.

According to the research of Bassi et al., in patients with amylase value in drains 5,000 U/L or less, in selected patients can suggest early drainage removal, early drain removal (3 days postoperative) may associated to a lower rate of pancreatic fistula and abdominal complications after pancreatic resections as opposed to drain removal according to standard policy (5 days postoperative or beyond) (17). When drain fluid amylase is low, early removal of the drainage tube may also effectively prevent the occurrence of a pancreatic fistula (18). In our study, an amylase level in the drainage fluid of <5,000 U/L was observed in 81.3% of patients. These patients underwent drainage tube removal after CT reexamination 5 days after surgery, which may be a reason for the low incidence of clinically relevant pancreatic fistula after longitudinal double U-shaped suture for anastomosis of the pancreatic duct and jejunal mucosa.

This study also has some limitations due to the retrospective design. A nonprospective randomized controlled trial that includes more patients and centers is needed to validate the rate of pancreatic fistula in this type of anastomosis.

## Conclusion

The laparoscopic-adapted technique we propose for performing the laparoscopic-adapted Blumgart anastomosis simplifies and facilitates the creation of the pancreaticojejunostomy in laparoscopic pancreaticoduodenectomy. The rates of clinically relevant postoperative pancreatic fistula are comparable with those obtained by open surgery, and length of stay are reduced in the laparoscopic pancreaticoduodenectomy. As a result, the use of laparoscopic-adapted Blumgart anastomosis may contribute to a significant reduction in the mean postoperative hospital stay in laparoscopic pancreaticoduodenectomy. However, the safety and feasibility of this technique should be verified by performing prospective randomized controlled trials at different institutions.

## Data Availability Statement

The original contributions generated for the study are included in the article/supplementary material, further inquiries can be directed to the corresponding author/s.

## Ethics Statement

The studies involving human participants were reviewed and approved by Weihai Cental Hospital. The patients/participants provided their written informed consent to participate in this study. Written informed consent was obtained from the individual(s) for the publication of any potentially identifiable images or data included in this article.

## Author Contributions

J-WL: conceptualization. Y-HY: data curation. P-JS: validation and writing of the original draft. X-JC: investigation. All authors contributed to the article and approved the submitted version.

## Conflict of Interest

The authors declare that the research was conducted in the absence of any commercial or financial relationships that could be construed as a potential conflict of interest.
